# Climatological, virological and sociological drivers of current and projected dengue fever outbreak dynamics in Sri Lanka

**DOI:** 10.1098/rsif.2020.0075

**Published:** 2020-06-03

**Authors:** Caroline E. Wagner, Milad Hooshyar, Rachel E. Baker, Wenchang Yang, Nimalan Arinaminpathy, Gabriel Vecchi, C. Jessica E. Metcalf, Amilcare Porporato, Bryan T. Grenfell

**Affiliations:** 1 Department of Ecology and Evolutionary Biology, Princeton University, Princeton, NJ 08544, USA; 2 Princeton Environmental Institute, Princeton University, Princeton, NJ 08544, USA; 3 Department of Civil and Environmental Engineering, Princeton University, Princeton, NJ 08544, USA; 4 Department of Geosciences, Princeton University, Princeton, NJ 08544, USA; 5 Department of Infectious Disease Epidemiology, Imperial College School of Medicine, London, UK; 6 Fogarty International Center, National Institutes of Health, Bethesda, MD 20892, USA

**Keywords:** dengue, time-series susceptible-infected-recovered, climate and disease, disease modelling, serotype

## Abstract

The largest ever Sri Lankan dengue outbreak of 2017 provides an opportunity for investigating the relative contributions of climatological, epidemiological and sociological drivers on the epidemic patterns of this clinically important vector-borne disease. To do so, we develop a climatologically driven disease transmission framework for dengue virus using spatially resolved temperature and precipitation data as well as the time-series susceptible-infected-recovered (SIR) model. From this framework, we first demonstrate that the distinct climatological patterns encountered across the island play an important role in establishing the typical yearly temporal dynamics of dengue, but alone are unable to account for the epidemic case numbers observed in Sri Lanka during 2017. Using a simplified two-strain SIR model, we demonstrate that the re-introduction of a dengue virus serotype that had been largely absent from the island in previous years may have played an important role in driving the epidemic, and provide a discussion of the possible roles for extreme weather events and human mobility patterns on the outbreak dynamics. Lastly, we provide estimates for the future burden of dengue across Sri Lanka using the Coupled Model Intercomparison Phase 5 climate projections. Critically, we demonstrate that climatological and serological factors can act synergistically to yield greater projected case numbers than would be expected from the presence of a single driver alone. Altogether, this work provides a holistic framework for teasing apart and analysing the various complex drivers of vector-borne disease outbreak dynamics.

## 1. Introduction

Dengue is a vector-borne disease with major clinical significance, accounting for 96 million apparent infections per year among inhabitants of 128 countries and an at-risk population of nearly four billion people [1]. Unlike many human pathogens, dengue virus (DENV) exists in only a small number (four) of phylogenetically and antigenically distinct primary groups [2]. Infection with one DENV strain confers life-long immunity to that particular strain, but not to the other three [2]. From an epidemiological perspective, these infection dynamics are particularly important owing to the increased risk of developing severe dengue upon secondary infection [3].

Dengue virus is spread primarily by the mosquito vector *Aedes aegypti* [4]. In brief, after taking a blood meal from an infected host (human or animal), a susceptible mosquito becomes infectious and able to transmit DENV to future hosts following a delay time known as the extrinsic incubation period (EIP) [5]. The EIP is dependent on the environmental temperature, along with the life cycle duration and survival of the mosquito [5,6]. In addition, precipitation and the local hydrological conditions can affect the quantity of pooled surface water, which serves as the breeding sites in which mosquitoes deposit their eggs. In particular, for dengue, *Aedes* mosquitoes are primarily container breeders (natural and artificial) and thrive in both clean and organically rich water [7]. Consequently, climate variables interact in complex ways with the biological parameters of the vector to determine not only the viable geographical range for the transmission of the disease [1,6,8] but also the timing and seasonality of infection in regions where the disease is endemic.

**Figure 1.**
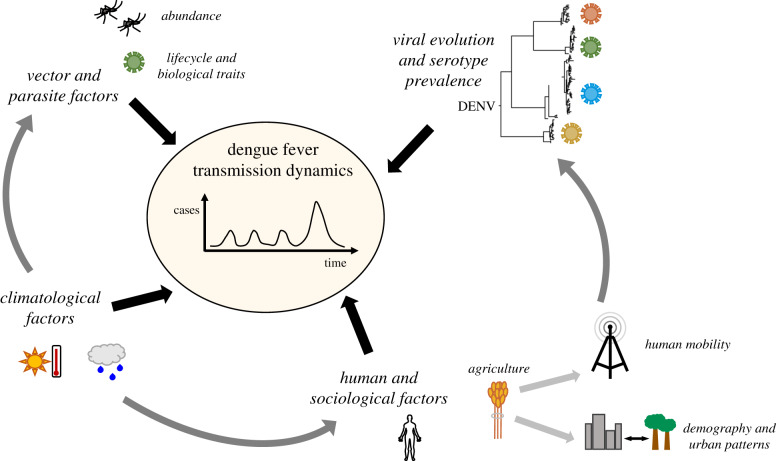
Schematic depicting the various potential drivers of the transmission dynamics of dengue as well as the relationships between these drivers. The depicted phylogeny of DENV is from [18].

In 2017, Sri Lanka experienced its largest recorded dengue outbreak, with 186 101 patient hospitalizations reported [9]. DENV has been endemic in Sri Lanka since the mid-1960s, and the four primary virus serotypes have been co-circulating there for more than three decades [7]. Furthermore, topographical influences as well as the seasonality of the monsoon cycles result in highly variable rainfall characteristics across the compact island nation [10], providing a unique opportunity to analyse localized interactions between climate and disease transmission. Indeed, several studies have determined a role for climatological drivers in the temporal dynamics of dengue incidence in Sri Lanka [11,12] (and more broadly [1,13–15]). In Sri Lanka, a variety of other factors have also been implicated in the epidemic and transmission dynamics of dengue, including changes in viral serotype [9], high population densities in affected areas [16], and poor hygiene conditions in urban environments [17]. This complex dynamical network of potential drivers is depicted schematically in figure 1. Recent work related to a dengue epidemic in Guangzhou, China has demonstrated that dengue epidemic periods in geographical regions with inter-annual transmission variability can serve as important case studies for assessing the relative importance of different drivers on DENV dynamics [19].

In this work, we systematically assess potential roles for climatological drivers including seasonal patterns in average temperature and precipitation as well as extreme weather events, epidemiological drivers including serotype change, and sociological drivers including human movement patterns in response to a severe drought, on the outbreak dynamics of dengue in Sri Lanka. We consider four distinct time periods in this analysis: the years 2010–2015 when endemic transmission patterns persisted, the transition year of 2016, the epidemic year of 2017, and finally future transmission patterns. We find that although seasonal climatological conditions play an important role in establishing monthly dengue transmission patterns in Sri Lanka, they alone are unable to account for the severity of the 2017 epidemic. Using a simplified two-strain susceptible-infected-recovered (SIR) model, we show that the re-introduction of a DENV serotype may have played an important role in driving the epidemic, and that in combination, climatological and serological drivers can act synergistically to yield greater projected case numbers than the presence of a single driver alone.

## 2. Results and Discussion

**Figure 2.**
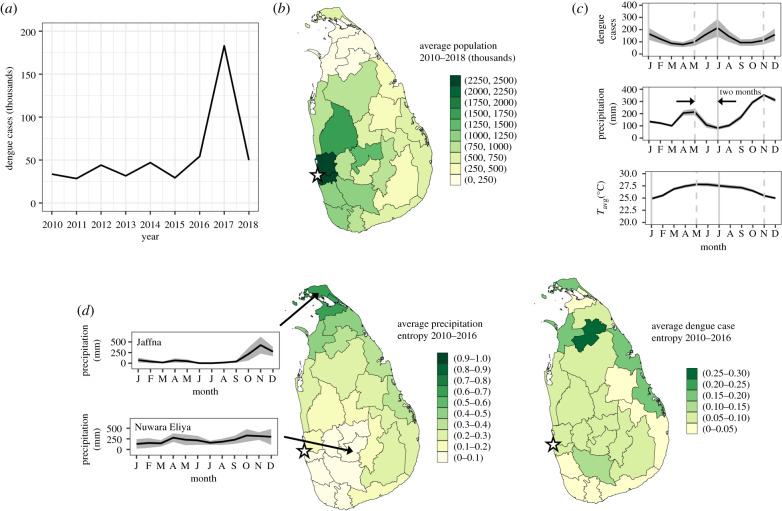
Dengue epidemiological data and relevant demographic and climatological information for Sri Lanka. (*a*) Annualized reported dengue cases across all 25 districts of Sri Lanka for the years 2010–2018. (*b*) Population of each district (in thousands) averaged over the years 2010–2018. (*c*) Mean monthly reported dengue cases (top panel), precipitation (middle panel), and average temperature (bottom panel) between the years 2010 and 2016 averaged across all districts (solid line). The shaded areas indicate the standard errors of the district means. Dashed grey lines in all charts are aligned with local maxima in precipitation, while the solid grey lines are aligned with the peaks in dengue cases. Together, these illustrate a two month lag between peak rainfall and peak dengue incidence. (*d*) Monthly entropy metric S by district for precipitation (centre panel) and dengue cases (right panel) from 2010 to 2016. Darker colours indicate districts with greater seasonal variability. Time series of monthly averaged precipitation (solid line) and standard deviation across years (shaded regions) for this same time period corresponding to the districts with the minimum (Nuwara Eliya, S=0.05), and maximum (Jaffna, S=0.67) precipitation entropy are also shown in the left panel. The approximate location of the national capital city of Colombo is indicated by a star in (*b*) and in the centre and right panels of (*d*).

In figure 2, an overview of the dengue incidence time series as well as relevant demographic and climatological information for Sri Lanka is provided. Dengue case data at a national level between the years 2010 and 2018 is plotted in figure 2*a*, showing a striking peak in case numbers in 2017. Population levels by district, averaged over the years 2010–2018, are presented in figure 2*b*.

In addition to substantial demographic variation by district, the tropical monsoon climate of Sri Lanka is characterized by elevated minimally varying average temperatures as well as highly variable rainfall characteristics [10]. Two precipitation-based climate zones can be distinguished within the island: the wet zone lying to the west of the central highlands, and the relatively drier zone lying to the east and north [10]. In general, the wet zone is exposed to the southwest monsoon from the months of May through to September as well as the first intermonsoon (lasting from March through to April), while the dry zone experiences substantial rainfall during the second intermonsoon (October through to November) and the northeast monsoon of December through to February [10].

In figure 2*c*,*d*, these climatic features for the pre-epidemic years of 2010–2016 are summarized and contrasted with the seasonality of dengue cases. In figure 2*c*, the mean monthly number of dengue cases, precipitation, and average temperature between the years 2010 and 2016 averaged across all districts are plotted as the solid lines, with the shaded areas indicating the standard error across districts. One immediately apparent feature is the relative uniformity of temperature both temporally over the year and across districts, as opposed to the clear seasonality in both precipitation and dengue cases, with larger standard errors indicative of greater variation between districts. Furthermore, two peaks in precipitation and dengue can be distinguished at this macroscopic level, with the former leading the latter by a period of two months.

In order to explore regional variation in seasonality of precipitation and dengue, a monthly relative entropy metric S [20,21] is calculated by district for these same data spanning the years 2010–2016, where

2.1 $$S=\sum_{m=1}^{M}x_{m}\log\left(Mx_{m}\right).$$

In equation (2.1), *M* = 12 is the number of months in the year and *x*_*m*_ is the monthly value of the parameter of interest divided by the mean annual value. Values of S→0 indicate minimal seasonal variation, i.e. more constant values of the parameter of interest throughout the year.

In the centre panel of figure 2*d*, the entropy metric S corresponding to the monthly mean precipitation between the years 2010 and 2016 is presented for each district. A notable geographical trend is observed, with low levels of precipitation entropy, or minimal seasonality, in the wet zone or southwest region of the country, and increasing precipitation entropy, or increasing seasonality, towards the northeast and the climatic drier zone. To illustrate the precipitation patterns corresponding to these values of S, time series of mean monthly precipitation from 2010 to 2016 with standard deviations across years indicated corresponding to the districts with the minimum (Nuwara Eliya, S=0.05), and maximum (Jaffna, S=0.67) precipitation entropy are shown in the left-hand panel. In the right-hand panel of figure 2*d*, this entropy calculation is repeated for mean monthly dengue case incidence between the years 2010 and 2016, and plotted for each district. Although less clear than that for precipitation, a similar trend of increasing seasonality in dengue case numbers is observed towards the northeast drier zone of Sri Lanka.

Motivated by the apparent correlation between precipitation and dengue cases observed in figure 2*d* as well as previous evidence of a role for climatological and other factors in establishing the temporal dynamics of dengue in the region [11,12], we analyse these epidemiological data within the framework of various disease drivers during four distinct periods: the years 2010–2015 when endemic transmission patterns persisted, the transition year of 2016, the epidemic year of 2017, as well as future years extending until 2099. We develop and use a climatologically informed time-series susceptible-infected-recovered (TSIR) model [22], in which temperature and precipitation levels or approximated serotype susceptibility play a role in determining either the dynamical transition of individuals within a given population from the susceptible to the infected category or the relative sizes of these subpopulations. The details of the development of this model are provided in the Material and methods section.

### 2.1. 2010–2015: endemic dynamics

**Figure 3.**
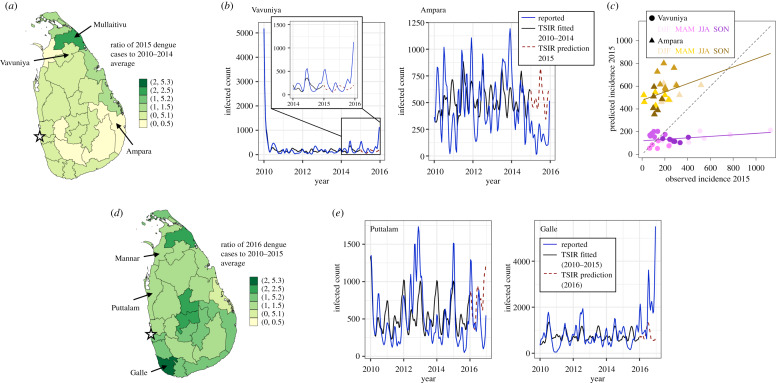
Epidemiological characterization and TSIR model fits for the periods of endemic (2010– 2015) and transitional (2016) dengue dynamics. (*a*) Ratio of reported dengue cases in 2015 to the average case numbers from 2010 to 2014 by district. The districts included in the panel regression model for which this ratio deviates the most (Ampara), and least (Vavuniya) from unity are indicated with arrows. The approximate location of the national capital city of Colombo is indicated by a star. (*b*) Dengue cases from 2010 to 2015, adjusted by the reporting rate *ρ* obtained from the TSIR model fitted to the data from the years 2010 to 2014 (solid blue line), TSIR model fitted to these data from 2010 to 2014 (solid black line), and TSIR predictions for the number of infected individuals in 2015 (dashed red line) for the districts of Vavuniya and Ampara. For clarity, these data for the years 2014 and 2015 for the district of Vavuniya are magnified and presented as an inset in the left-hand panel. (*c*) Scatter plot of dengue cases in 2015 adjusted by the reporting rate *ρ* obtained from the TSIR model fitted to the data from the years 2010 to 2014 (solid blue curve of (*b*)) versus the TSIR predictions for the number of infected individuals in 2015 (dashed red line of (*b*)) for the districts of Vavuniya (purple colour scheme, circles) and Ampara (yellow colour scheme, triangles). The colour intensity corresponds to season, with the lightest hues corresponding to winter months (December, January, February (DJF)), then spring months (March, April, May (MAM)), summer months (June, July August (JJA)), and the darkest hues corresponding to autumn months (September, October, November (SON)). The solid lines of corresponding colour scheme denote linear regression fits to these district-level data. The dashed black line corresponds to parity between the observed and predicted values. (*d*) Ratio of reported dengue cases in 2016 to the average case numbers from 2010 to 2015 by district. The districts included in the panel regression model for which this ratio deviates the most (Galle), and least (Puttalam) from unity are indicated with arrows. The approximate location of the national capital city of Colombo is indicated by a star. (*e*) Dengue cases from 2010 to 2016, adjusted by the reporting rate *ρ* obtained from the TSIR model fitted to the data from the years 2010 to 2015 (solid blue line), TSIR model fitted to these data from 2010 to 2015 (solid black line), and TSIR predictions for the number of infected individuals in 2016 (dashed red line) for the districts of Puttalam and Galle.

We begin by studying the ability of the TSIR model [22] to capture the more regular endemic dynamics of dengue incidence from 2010 to 2015, prior to the dynamical transitions characterizing the epidemic year of 2017. This relative case regularity is evident in figure 3*a*, where the ratio of dengue cases by district in 2015 to the average of those reported in the years 2010 to 2014 (*C*_2015_/*C*_2010–2014_) is plotted and found to range between values of 0.36 ≤ *C*_2015_/*C*_2010–2014_ ≤ 2.06.

In order to evaluate the predictive ability of the TSIR model during these endemic years, we train the model on district-level case data from 2010 to 2014, and assess its ability to predict the 2015 case numbers. In figure 3*b*, the number of cases from 2010 to 2015 adjusted by the reporting rate *ρ* obtained from the TSIR model fitted to the case data from 2010 to 2014, the TSIR fitted from 2010 to 2014, and the predicted case numbers for 2015 are plotted as the solid blue, solid black and dashed red curves, respectively, for the districts in which the ratio *C*_2015_/*C*_2010–2014_ deviates the most (Ampara, *C*_2015_/*C*_2010–2014_ = 0.36), and least (Vavuniya, *C*_2015_/*C*_2010–2014_ = 0.99) from unity. Here, we consider only districts included in the climatological regression model, thus excluding (for example) Mullaitivu, which has a ratio of *C*_2015_/*C*_2010–2014_ = 2.06 (as described in the Material and methods section, Mullaitivu is excluded from this analysis as an outlier, consistent with its exclusion from the climatological regression model). As can be seen, the TSIR model produces a satisfactory prediction of the 2015 case numbers in both districts, with cyclic seasonal trends well-captured despite disagreement in quantitative values. This is also summarized in the scatter plot of the number of dengue cases in 2015 adjusted by the reporting rate *ρ* obtained from the TSIR model fitted to the data from the years 2010 to 2014 versus the TSIR predictions for the number of infected individuals in 2015 for the districts of Vavuniya (purple colour scheme, circles) and Ampara (yellow colour scheme, triangles) presented in figure 3*c*. These data are further organized by season, with the lightest hues corresponding to winter months (December, January, February (DJF)), then spring months (March, April, May (MAM)), summer months (June, July August (JJA)) and finally the darkest hues correspond to autumn months (September, October, November (SON)). In Vavuniya, the observed and predicted case numbers agree well (lie near the dotted black line indicating parity) apart from in the winter months when the observed case numbers increase sharply. By contrast, the case numbers are over-predicted in Ampara for all seasons.

### 2.2. 2016: transitional dynamics

In certain districts, the onset of the epidemic period occurred as early as partway through 2016, thus warranting a separate consideration of this transitional year. To illustrate this, in figure 3*d*, the ratio *C*_2016_/*C*_2010–2015_ of dengue cases by district in 2016 to the average of those reported in the years 2010–2015 is shown. As can be seen, in contrast with figure 3*a*, the value of this ratio is now greater than unity in nearly all districts. As in §2.1., we next assess the ability of the TSIR model to predict the 2016 case numbers in the district for which *C*_2016_/*C*_2010–2015_ deviates the most (Galle, *C*_2016_/*C*_2010–2015_ = 2.76), and least (Puttalam, *C*_2016_/*C*_2010–2015_ = 1.04) from unity. Here, we consider only districts included in the climatological regression model, thus excluding (for example) Mannar, which has a ratio of *C*_2015_/*C*_2010–2014_ = 1.00 (as described in the Material and methods section, Mannar is excluded from this analysis as an outlier, consistent with its exclusion from the climatological regression model). In figure 3*e*, the number of cases from 2010 to 2016 adjusted by the reporting rate *ρ* obtained from the TSIR model fitted to the case data from 2010 to 2015, the TSIR fitted from 2010 to 2015, and the predicted case numbers for 2016 are plotted as the solid blue, solid black, and dashed red curves, respectively, for the districts of Puttalam and Galle. As can be seen, for the district of Puttalam where the epidemic had not begun by the end of 2016, the TSIR model produces a satisfactory prediction of the 2016 case numbers, with the climatological seasonal cycle well-captured despite disagreement in quantitative values. Conversely, in Galle, where the epidemic begins midway through the year 2016, there is substantial disagreement between the prediction of the TSIR model and the reported case numbers.

### 2.3. 2017: epidemic dynamics

**Figure 4.**
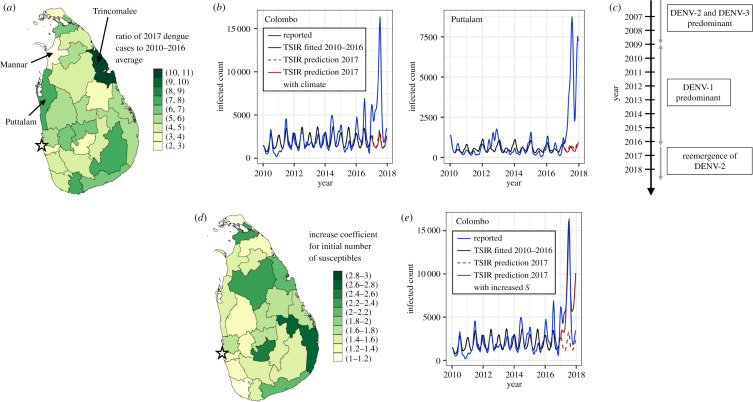
Epidemiological characterization and TSIR model fits for the epidemic dengue year of 2017. (*a*) Ratio of reported dengue cases in 2017 to the average case numbers from 2010 to 2016 by district. (*b*) Dengue cases from 2010 to 2017 adjusted by the reporting rate *ρ* obtained from the TSIR model fitted to the case data from 2010 to 2016 (solid blue line), TSIR model fitted from 2010 to 2016 (solid black line), and TSIR predictions for 2017 (dashed red line) for the districts of Puttalam and Colombo. The solid red line corresponds to the TSIR predictions for 2017 explicitly accounting for the climate of 2017 by deriving the transmission rate from the panel regression model. (*c*) Timeline of dominant dengue serotypes in Sri Lanka based on clinical and laboratory features reported in [9]. (*d*) Factorial increase in the number of susceptible individuals reconstructed by the TSIR model by district for the end of 2016 that is required in order to adequately capture the maximum number of reported dengue cases for 2017. (*e*) Dengue cases from 2010 to 2017 adjusted by the reporting rate *ρ* obtained from the TSIR model fitted to the case data from 2010 to 2016 (solid blue line), TSIR model fitted from 2010 to 2016 (solid black line), and TSIR predictions for 2017 (dashed red line) for the district of Colombo. The solid red line corresponds to the TSIR predictions for 2017 when the initial number of susceptible individuals is increased by the factor indicated in (*d*). The approximate location of the national capital city of Colombo is indicated by a star in (*a*) and (*d*).

Next, we consider the dynamics of the epidemic year of 2017. As can be seen in figure 4*a*, dengue incidence in 2017 was higher in all districts compared to the previous 7 years, ranging from a 10.7-fold increase in the district of Trincomalee to a 2.3-fold increase in the district of Mannar. In this section, we seek to explore the possible drivers that may have contributed to these epidemic dynamics.

#### 2.3.1. Climatological drivers

We begin by testing the role of climate, and repeat the exercise from figure 3*b*,*e*, this time training the model on data from 2010 to 2016 and obtaining predictions for the 2017 case numbers for the districts of Puttalam (where the TSIR prediction for 2016 was satisfactory) as well as the district of Colombo which contains the national capital city of the same name. These results are shown in figure 4*b*. We additionally perform the model prediction for 2017 using values for the transmission rate *β* derived from the climatological regression model (see the Material and methods section) that explicitly accounts for the climate of 2017, and plot these results as the solid red curve in figure 4*b*. As can be seen, although the timing of dengue case seasonality is relatively well captured by the TSIR predictions in both districts, even when the climate from 2017 is explicitly accounted for, the reported case numbers are substantially under-predicted. Consequently, it would appear that although climate plays an important role in establishing the timing of annual dengue transmission patterns in Sri Lanka, the severity of the 2017 epidemic cannot be explained by an anomalous climate that year. Figure 3*b* is reproduced for all districts in the electronic supplementary material, figure S8.

#### 2.3.2. Virological drivers

As illustrated in figure 4*c*, according to hospital surveillance reports, DENV1 was the predominant serotype in circulation in Sri Lanka from 2009 until mid-2016, during which time DENV2 and DENV3 were not detected [9]. DENV2 and DENV3 were the predominant serotypes in circulation prior to 2009, and indeed all four virus serotypes have been co-circulating in Sri Lanka for more than three decades [7,9]. In mid-2016, the re-emergence of DENV2 coincided with the onset of the epidemic [9]. Sequencing studies of the 2016 Sri Lankan DENV2 strain obtained from patients who were treated for acute dengue infection at the National Institute of Infectious Disease confirmed it to be from the same clade as those circulating in 2015–2016 in Singapore, and indicated that it shared a common ancestor with the 2014 Malaysian strains [9]. In fact, these studies found that the 2016 Sri Lankan DENV2 strain was genetically more distant from the DENV2 strains that circulated in Sri Lanka from 1981 to 2004 than these Singaporean and Malaysian strains [9], suggesting case importation. The phylogeny of DENV from [18] is included in the electronic supplementary material, figure S1*a*.

In order to estimate the possible role for serotype re-emergence on the epidemic dynamics, we model the predicted effect of an effective increase in population-level susceptibility at the start of the epidemic. This increase can be interpreted as representing individuals in the population who had previously been infected with DENV1 only (and were hence still susceptible to DENV2 infection), or an increase in the severity of secondary DENV infections relative to primary ones [2] following replacement of the dominant strain. Indeed, over short time periods, the TSIR yields similar predicted increases in case numbers when either the initial number of susceptible individuals or the transmission rate *β* is increased. It is worth noting, however, that in one clinical report [9], all patients with acute dengue infection recruited after January 2017 by the National Institute of Infectious Disease of Sri Lanka were infected with DENV2, and that high levels of patients presenting with both DENV1 (pre-epidemic) and DENV2 (post-epidemic) were experiencing secondary infections. Whether this extends to the district-level data used in this paper is unknown. We emphasize then that with these clinical observations in mind, the analysis in this section serves as a reasonable starting point for considering the role of virological drivers given the absence of serotype information. Further caveats are discussed in §2.5.

In figure 4*d*, the multiplicative factor on the initial value of susceptible individuals at the start of 2017 that minimizes the difference in the peak number of reported and predicted cases in 2017 is plotted for each district, and ranges between a value of 1.11 and 2.61. In figure 4*e*, the number of cases from 2010 to 2017 adjusted by the reporting rate *ρ* obtained from the TSIR model fitted to the case data from 2010 to 2016, the TSIR fitted from 2010 to 2016, and the predicted case numbers for 2017 are plotted as the solid blue, solid black and dashed red curves, respectively, for the district of Colombo. Additionally, we plot the TSIR prediction for 2017 following re-initialization of the initial number of susceptible individuals by the multiplicative factor obtained in figure 4*d* as the solid red curve.

In order to contextualize these determined increases in the number of susceptible individuals, we consider the simplified scenario of a two-strain system, in which we assume that a single DENV serotype has achieved steady-state transmission dynamics prior to the arrival by exogenous importation of a new viral strain. As described above, this approximation is consistent with sequencing results of the genetic origins of the 2016 Sri Lankan DENV2 strain. However, this scenario ignores the complexities of cross-immunity associated with the long-term co-circulation of all four DENV strains in Sri Lanka. Further discussion of the caveats of this two-strain system are discussed in §2.5. Nevertheless, in this simplified scenario, the number of individuals susceptible to the newly imported strain and/or the previously circulating one would exceed the steady-state number of susceptible individuals to the previously established strain only. A schematic of this simplified two-strain system based on that developed in [23] is shown in the electronic supplementary material, figure S1*b* and the mathematical details are described in the Material and methods section.

At the time of importation of serotype 2 infections, we approximate the actual number of susceptible individuals *S*′ as

2.2 $$S^{\prime }\approx S+\frac{R_{1}\rho_{2}N}{I_{1}}\approx S^{*}\left[1+\frac{\rho_{2}\gamma \beta }{\mu (\gamma +\mu )}\right],$$

where *S* is the number of individuals susceptible to infection by both strains, S* = *N*/*β*(*γ* + *μ*) is the steady-state value of this quantity when a single strain is in circulation (see the Material and methods section), *I*_1_ and *R*_1_ are, respectively, the number of individuals presently infected with or recovered from serotype 1 infections only, and *N* is the total number of individuals. Furthermore, *μ*, *γ*, *β* and *ρ*_2_ denote the rates of birth, death, transmission and importation of exogenous infections of serotype 2, respectively. The analytic expression for the factorial increase in the susceptible population in equation (2.2) allows us to consider the magnitude of the importation rate of infections of the exogenous serotypes that would yield the results found in figure 4*d*. The multiplicative factor for the number of susceptible individuals predicted for the district of Colombo is *S*′ = 1.27S*. Furthermore, given a recovery rate for dengue of *γ* = 1 biweek^−1^, an average crude birth rate of *μ* = 8.55 × 10^−4^ biweek^−1^ for the years 2010–2016, and a mean population-normalized value for the transmission rate reconstructed from the TSIR model fitted for the years 2010–2016 of *β*/*N* = 2.38 × 10^−6^ person^−1^ biweek^−1^, the predicted number of imported cases for the first biweek of 2017 in the district of Colombo is found to be *ρ*_2_ *N* = 96.9. Although the dengue dataset used in this work does not contain information regarding whether cases are autochthonous or imported, recent network models have been developed for predicting the number of imported dengue cases for any given airport [24].

#### 2.3.3. Other drivers

**Figure 5.**
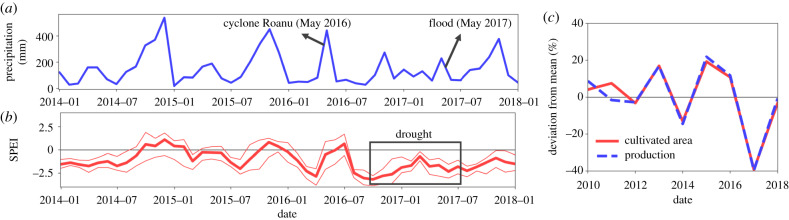
Characterization of national precipitation levels and agricultural production prior to, during, and after the dengue epidemic of 2017. (*a*) Spatial average of monthly precipitation across the country, with the timing of extreme climatic events indicated. (*b*) Three-month spatial average of the standardized precipitation-evapotranspiration index (SPEI) which quantifies drought severity. Large negative values correspond to more severe drought conditions. The thick red line corresponds to the average value, whereas the thinner lines show the spatial minimum and maximum values across the country. (*c*) Deviation of the total cultivated area and production of rice from the average value between the years 2010 and 2018.

Finally, it is worth noting a number of other factors that may have influenced the dynamical pattern of the 2017 dengue epidemic. Between the spring of 2016 and the summer of 2017, Sri Lanka experienced a series of extreme climatic events including both severe droughts and floods. As seen in figure 5*a* which shows the spatially averaged monthly precipitation across the country, cyclone Roanu hit the island in May 2016 causing severe flooding and landslides which led to over 200 individuals being reported dead or missing. Subsequently, the island experienced a severe drought owing to diminished winter precipitation in 2016. These drought conditions are illustrated in figure 5*b* by the mean, minimum and maximum spatially averaged values of the standardized precipitation-evapotranspiration index (SPEI) [25].

The 2017 drought had a severe impact on the agricultural sector, causing a 40% decrease in the cultivated area and production of rice as depicted in figure 5*c*. Economic theory and empirical research in a number of developing economies including Senegal have shown that increases in the ratio of urban-to-rural wages, possibly driven by decreases in agricultural output, provide the foundation of rural–urban migration [26,27], and news reports suggest that this phenomenon occurred in Sri Lanka during this period [28]. Changes in human social ecology, including population size and density and urbanization, have been implicated in altering the probabilities of infectious disease emergence and transmission by creating highways for ‘microbial traffic’ and providing opportunities for rapid dissemination of emerging infections [29]. These various factors suggest that in-depth studies into the effect of human mobility patterns on the dengue epidemic of 2017 may be warranted, such as those conducted using mobile phone data in Pakistan [30] and for rubella in Kenya [31]. It is nevertheless interesting to consider the magnitude of the population that would be required to relocate in order to achieve the fractional increases in the susceptible population calculated in §2.3.2. In the district of Colombo, for instance, a 27% increase in the susceptible population (i.e. a fractional increase of *S*′ = 1.27S*) corresponds to an influx of 256 000 susceptible individuals; approximately 11% of its population in 2017.

Lastly, we note that the length of the approximately 7.5 year period from 2009 until mid-2016 during which DENV1 was the primary serotype in circulation prior to the re-emergence of DENV2 is broadly consistent with the periodicity of serotype prevalence of 8–9 years determined both theoretically and from epidemiological data in [32], although shorter cycles of 3–5 years have also been observed in both data and simulations [2]. Depending on the modelling framework, a number of factors have been found to contribute to the maintenance of these multi-year cycles, including virological cross-protection [2] as well as the segregation of hosts via spatially arranged communities with the possibility of importation of infections via temporal human movements [32]. Therefore, although these complex multi-annual patterns are undoubtedly driven by a number of factors, it is possible that these periods of serotype prevalence define an upper bound on the allowed build-up of the susceptible cohort, beyond which the re-introduction of a non-predominant viral strain may result in the onset of an epidemic.

### 2.4. Projections for future transmission dynamics

As a final exercise, we use the climatological panel regression model developed for the years 2010–2016 in combination with the projections of average temperature and precipitation obtained from the multi-model ensemble Coupled Model Intercomparison Phase 5 (CMIP5) dataset under Representative Concentration Pathway 8.5 in order to study the potential future transmission dynamics of dengue in Sri Lanka.

**Figure 6.**
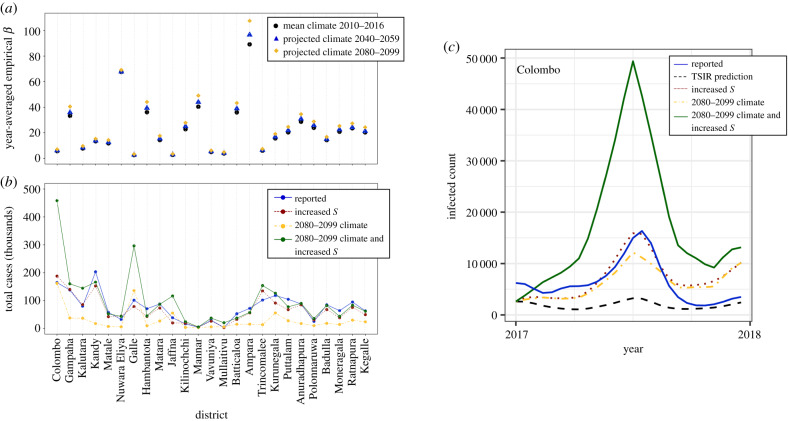
Effect of climate and serotype drivers on future dengue transmission dynamics. (*a*) Mean annual empirical dengue transmission rates by district obtained from the panel regression model assuming a linear dependence of log transmission on precipitation and a Brière functional form for average temperature. The present-day values obtained using the mean climate data from 2010 to 2016 are shown as black circles. Future mean transmission rates obtained from projections of average temperature and precipitation are shown for the time periods 2040–2059 (blue triangles) and 2080–2099 (yellow diamonds). (*b*) Total number of cases for the year 2017 by district under the conditions of: 2080–2099 climate (yellow circles and dashed-dotted yellow line), increased initial number of susceptible individuals to reflect serotype invasion (red circles and dashed red lines), and both 2080–2099 climate and increased initial number of susceptible individuals (green circles and solid green line). The actual case numbers for 2017 adjusted for the reporting rate *ρ* obtained from the TSIR model fitted to the case data from 2010 to 2016 are shown by the blue circles and solid blue lines. (*c*) Dengue cases for 2017 adjusted by the reporting rate *ρ* obtained from the TSIR model fitted to the case data from 2010 to 2016 (solid blue line) and TSIR predictions for the number of infected individuals for 2017 following training on 2010–2016 data only (dashed black line) for the district of Colombo. The TSIR predictions for 2017 under the conditions of increased initial number of susceptible individuals, use of the transmission rate derived from the 2080 to 2099 climate projection data, and both climate and serotype adjustments are shown as the dotted red, dashed-dotted yellow and solid green lines, respectively.

In figure 6*a*, the year-averaged empirical transmission rates *β* for the time periods 2040–2059 (blue triangles) and 2080–2099 (yellow diamonds) predicted by the climatological panel regression model are shown, and compared with the present-day levels obtained using average climate data from 2010 to 2016 (black circles). Although there is substantial variation across districts, the year-averaged value of *β* is predicted to increase approximately consistently with time, primarily as a result of projections of increasing average temperature during this time period (see the electronic supplementary material, figures S2 and S3 and the discussion in S6). Note that location-by-month and location-by-year dummies for the three districts that were excluded from the panel regression model (see the Material and methods section) were obtained by re-running the panel regression model with all districts included. The coefficients on the climate variables used for the transmission rate projections for these three districts, however, were set to those obtained by the regression model when these three districts were excluded. Furthermore, in obtaining the future values, it is important to note that only climatological variables were modified in the panel regression model and all location- and time-specific dummy variables were held constant.

In order to better interpret these predictions for the empirical transmission rate, we re-run the TSIR model for 2017, as was done in §2.3.1., using the projected values for the transmission rate obtained from the panel regression model with climate data for 2080–2099. Furthermore, because serotype invasion was found in §2.3.2. to be a potentially important driver of outbreak dynamics, we also consider the compound scenario in which the same climatological factors are considered and in addition the baseline number of susceptible individuals is increased to *S*′ (see equation (2.2)). Consequently, these simulations are relevant for considering the effect of altered climate variables and/or the introduction of a previously absent serotype when all other demographic variables including birth rates and population levels are held constant.

In figure 6*b*, the total number of cases for the year 2017 under these various scenarios are reported for each district. The blue circles and solid blue line correspond to the actual case numbers adjusted for the reporting rate *ρ* obtained from the TSIR model fitted to the case data from the years 2010 to 2016; the yellow circles and dashed-dotted yellow line correspond to the predicted case numbers when the transmission rate obtained from the panel regression model with 2080–2099 climate data is used; the red circles and dashed red lines correspond to the scenario of simulated serotype invasion shown in figure 4*e*, in which only the initial number of susceptible individuals is adjusted by the district-specific multiplicative factor reported in figure 4*d*; and the green circles and solid green line correspond to the scenario of both 2080–2099 climate and serotype invasion. Once again, substantial variation is observed across districts, but critical increases in case numbers under the compound climatological and serological driver scenario are observed in a number of districts, including the most populous district of Colombo in which the national capital city of the same name is located. In quantitative terms, in Colombo the total predicted case numbers under the compound driver scenario are 2.84-fold and 2.44-fold higher than those predicted by the scenarios of 2080–2099 climate and serotype invasion alone, respectively. Dengue incidence across the year is shown for Colombo in figure 6*c* under these same conditions, as well as the predictions of the TSIR model for 2017 following training on the 2010–2016 data only (the dashed black curve). Figure 6*c* is reproduced for all districts in the electronic supplementary material, figure S9.

### 2.5. Caveats

This work provides an interdisciplinary analysis of the potential drivers of dengue dynamics in Sri Lanka, and consequently it is important to explicitly mention a number of caveats that should be considered when interpreting the findings presented herein. Firstly, as described in the Material and methods section, the panel regression model assumes a Brière functional form for the average temperature in determining the empirical transmission rate. This was done to capture the non-monotonic thermal responses generally observed for mosquito and viral traits [33]. One immediate issue with this approach, however, is the shortage of data points on the decreasing portion of the Brière curve (see the electronic supplementary material, figure S5*c*). Although we lack observational data to populate this curve owing to the limited range of average temperatures contained within the regression panel, we hypothesize that above a critical value, increasing values of average temperature would correlate negatively with log-transmission rate. Indeed, several of the critical temperature values estimated in [33] lie above the range of average temperature values measured in our climatological dataset. Somewhat reassuringly, however, because the predicted future values of average temperature (see the electronic supplementary material, figure S2) generally lie below the critical thermal response temperature obtained for the panel regression model (see the Material and methods section), the assumption of either a Brière or linear functional form for average temperature yielded similar predictions for log-transmission rates. One interesting exception is the district of Nuwara Eliya, whose yearly average temperature values in the present day as well as projected future values generally lie below the value of *T*_min_ determined for the Brière function (see the electronic supplementary material, figure S2). The assumption of a Brière functional form for average temperature in the regression model consequently results in minimal projected changes in log transmission (figure 6*a*), while a much greater increase is projected when a linear functional form is used (electronic supplementary material, figure S6).

Significant variation across districts was obtained for the lag times between dengue case numbers and both average temperature and precipitation (see the electronic supplementary material, figure S4), and these values were also sensitive to the number of years of data considered by the panel regression model. In order to capture relevant long-term trends, we chose to develop the panel regression model using the maximum number of years of epidemiological data available prior to the epidemic year (2010–2016), and assumed a single lag value between the case data and climate variables for the entire country by selecting that which occurred most frequently across districts. Nevertheless, if more accurate dengue case predictions based on future climate projections for a specific district are desired, it may be more appropriate to use district-specific as opposed to national lag values for the climate variables, and to adjust the years considered in the panel regression model accordingly.

Furthermore, given the absence of serotype data, it is important to carefully consider the limitations of the serologically-motivated analysis in §§2.3.2. and 2.4. As previously mentioned, modelling an effective increase in population-level susceptibility or an increase in the transmission rate *β* yield similar increases in predicted case numbers using the TSIR model over short-time periods. These scenarios may represent additional cases owing to secondary infections and potential increases in the severity of secondary DENV infections relative to primary ones [2]. While both of these scenarios are relevant to multi-strain pathogenic systems, they highly simplify the reality of DENV infections in Sri Lanka, which include endemic co-circulation of four viral strains and temporary periods of cross-immunity between heterologous strains [2]. There is evidence, however, that for multi-year periods, one DENV strain dominates and is eventually replaced by another serotype (see case data from Thailand [2] and Puerto Rico [32], for instance). In that sense, at specific periods in time corresponding to strain replacement, it may be justified to think about DENV infection as a two-strain system, although in order to model long-term serotype interaction dynamics this approach is clearly not justified. Therefore, given the absence of serotype-specific case data but clinical evidence of serotype invasion, our approach provides a starting point for analysis despite its numerous limitations. Furthermore, in our analytical consideration of the potential magnitude of case importation, it was assumed that *R*_0_ was identical for primary and secondary dengue infections. Although this may be correct to first order, it is certainly possible that transmission rates may vary between serotypes. Additionally, in the derivation of the multiplicative increase factor for the number of susceptible individuals (see the Material and methods section and equation (2.2)), it was assumed that the number of imported cases was much smaller than the number of autochthonous ones. Although this assumption may hold true in regions where dengue is endemic, it may not be valid in non-endemic areas.

Finally, as described in §2.4., projections of future transmission rates and dengue case numbers were obtained by considering modifications to the climatological and serological drivers of the TSIR model only. In other words, inherent in these projections are assumptions of unchanging location- and time-specific dummy variables in the panel regression model, as well as unchanging demographic patterns including birth rates and population levels in the TSIR model. Consequently, our simulations are designed to examine the effect of a changing climate and serotype invasion on dengue transmission *all other things being equal*, and care should be taken when basing claims about future dynamical trends on these results.

## 3. Conclusion

Dengue is a geographically widespread disease of major clinical significance, whose transmission dynamics are well known to be modulated by a number of drivers including the local climate, human ecology and behaviour, and biological interactions between the four distinct primary serotypes. Particularly in light of the absence of vaccines or antivirals for this disease, there remain important opportunities to analyse the transmission dynamics of dengue in a holistic fashion by integrating these distinct and complex drivers. In this work, we do so in the context of the largest ever dengue epidemic in Sri Lanka during 2017, specifically considering climate, serotype invasion, and human mobility as epidemic drivers.

Sri Lanka provides a particularly powerful location for studying the effect of climate on dengue transmission as a result of highly variable rainfall patterns within the island. By developing a climatologically informed TSIR model, we found, in agreement with previous work, that seasonal climatological conditions play an important role in establishing yearly dengue transmission patterns in Sri Lanka, however they were unable to independently account for the severity of the 2017 epidemic. Motivated by hospital reports of a shift in predominant serotype from DENV1 to DENV2 midway through 2016, we modified a simplistic two-strain model for dengue transmission and demonstrated that increasing the number of susceptible individuals reconstructed by the TSIR model at the start of the epidemic year in order to account for secondary infections, was sufficient to capture the epidemic case numbers of 2017. Additionally, evidence of significantly reduced agricultural output during 2017 as a result of a series of severe climatic events suggests that a careful analysis of internal migration during this year as a driver of increased dengue transmission may be warranted. Critically, our simulations also suggest that the combination of climatological changes and serotype invasion can create compound effects on dengue transmission, resulting in even greater predicted case numbers than when only a single driver is assumed to be present. This possibility of synergistic interactions between disease drivers further reinforces the need to consider the epidemiology of dengue holistically.

To conclude, a number of important studies have demonstrated a role for the various drivers presented in figure 1 in the outbreak dynamics of dengue [1,2,13–15,23,30–32]. Here, we establish a holistic framework for teasing apart and analysing these various drivers, thus bringing together much of this prior work in the context of the 2017 Sri Lankan dengue epidemic. Such a multifaceted analysis may prove to be increasingly important in the face of changes in human ecology driven by urbanization and development, as well as climate change.

## 4. Material and methods

### 4.1. Dengue case data

Dengue case data were obtained from the publicly available disease surveillance dataset provided online by the Epidemiology Unit of the Sri Lankan Ministry of Health [34]. Cases are defined based on the presentation of a required number of symptoms. Cases are reported based on three types of surveillance: passive surveillance (submission of a notification card to a Medical Officer of Health), sentinel site surveillance (cases entered into web-based system by hospital officer), and special surveillance (used to identify the dynamics of dengue fever and dengue haemorrhagic fever). Monthly case data ranging from January 2010 to December 2018 were recorded for each of the 25 districts.

### 4.2. Demographic data

District-level demographic data for Sri Lanka were obtained from the CEIC database [35] as well as the Department of Census and Statistics of Sri Lanka [36].

### 4.3. Climatological data

Daily surface air temperature data were obtained from the ERA-5 global atmospheric reanalysis dataset [37]. The native 0.25 degree gridded dataset was interpolated bilinearly to a 0.05 degree grid. Temperature data for a given district were obtained by averaging the values at all grid points located within its geographical limits.

Daily precipitation data were obtained from the Climate Hazards Group InfraRed Precipitation with Station (CHIRPS) 0.05 degree gridded dataset [38,39]. As with temperature, precipitation data for a given district was obtained by averaging the values at all grid points located within its geographical limits.

Monthly SPEI data with a three-month time scale were obtained from the 1 degree gridded SPEI Global Drought Monitor dataset [40]. The time series of SPEI was computed by averaging SPEI values of all pixels within the island boundaries.

Climate projection data were obtained from the CMIP5 multi-model means dataset MMM-v2 which averages over 38 models under Representative Concentration Pathway 8.5 [41]. The native 2 degree gridded dataset was interpolated bilinearly to a 0.05 degree grid. As for the reanalysis data, the value for a given district was obtained by averaging the values at all grid points located within its geographical limits. Projections for temperature *T*_fut_(*i*, *m*) during month *m* in district *i* in the future period of interest were obtained using the formula

4.1 $$T_{\mathrm{fut}}(i,m)=T_{\mathrm{pres}}(i,m)+(T_{\mathrm{CMIP}5\mathrm{fut}}(i,m)-T_{\mathrm{CMIP}5\mathrm{pres}}(i,m)).$$

In equation (4.1), *T*_pres_(*i*, *m*) and *T*_CMIP5pres_(*i*, *m*) are the reanalysis and CMIP5 model present-day values for surface air temperature, where the present day is taken to be the average value over the 20 year period of 1995–2014. Similarly, *T*_CMIP5fut_(*i*, *m*) is the CMIP5 model value of surface air temperature for the future period of interest.

Projections for precipitation *P*_fut_(*i*, *m*) during month *m* in district *i* in the future period of interest were obtained using the formula

4.2 $$P_{\mathrm{fut}}(i,m)=P_{\mathrm{pres}}(i,m)*(P_{\mathrm{CMIP}5\mathrm{fut}}(i,m)/P_{\mathrm{CMIP}5\mathrm{pres}}(i,m)).$$

In equation (4.2), *P*_pres_(*i*, *m*) and *P*_CMIP5pres_(*i*, *m*) are the reanalysis and CMIP5 model present-day values for precipitation, where the present day is taken to be the average value over the 20 year period of 1995–2014. Similarly, *P*_CMIP5fut_(*i*, *m*) is the CMIP5 model value of precipitation for the future period of interest.

District-level yearly plots of average temperature and precipitation corresponding to the present day (2010–2016) mean as well as the 2040–2059 and 2080–2099 projections are provided in the electronic supplementary material, figures S2 and S3, respectively.

### 4.4. Agricultural data

Data for annual district level cultivated area and rice production were obtained from the Department of Census and Statistics of Sri Lanka [36]. The data included two cultivation seasons: Maha from September to March, and Yala from May to August. Annual values were obtained by summing the cultivated area and production data for the two cultivation seasons every year.

### 4.5. Development of the climatologically informed transmission model

SIR models form a family of simple mass-action epidemic models in which individuals within an assumed well-mixed population transition between the categories of susceptible (S), infected (I) or recovered (R) depending on their disease status to a given pathogen [42]. In this framework, transitions between categories are governed by the demographic processes of birth (which feeds the population of susceptible individuals) and death, as well as the disease transmission rate *β* driving susceptible individuals into the infected category and the recovery rate *γ* at which individuals move from the infected to recovered class. For both directly- and vector-transmitted diseases, the transmission rate *β* is frequently seasonal, reflecting yearly variation in disease drivers such as climatic conditions or demographic considerations such as the timing of school terms [22].

The stochastic, discrete-time TSIR model [22] provides a computationally inexpensive, highly tractable method for calibrating these seasonally varying, continuous time SIR models against time-series case data [42,43], and has previously been used to model the temporal dynamics of DENV [2,8]. From time series of disease case counts, births and total population numbers, the TSIR model constructs estimates for the susceptible population and transmission rate at discrete time points corresponding to the generation time of the disease in question. In this work, numerical implementation of the TSIR model was performed using the R package tsiR [42]. Following [43], we seek to formulate a regression model to estimate the effect of the climatological variables of average temperature and precipitation on the transmission rate *β* constructed by the TSIR model.

#### 4.5.1. Time-series susceptible-infected-recovered model fitting and parameter details

Inputs for the TSIR model included district-level dengue case data (described in §4.1.) as well as district-level population levels and births for the relevant time periods (obtained from the sources described in §4.2.). A linear regression model was used for susceptible reconstruction, resulting in a constant reporting rate *ρ* for each fit performed. The values of *ρ* obtained from the TSIR model fitted to the case data from 2010 to 2016 for each district are plotted in the electronic supplementary material, figure S7. The mixing parameter *α* (described in further detail in §4.5.2. below) was left flexible and hence was fitted by the function. The generalized linear model family chosen to estimate *α* and the transmission rate *β* was Poisson with a log link [42].

#### 4.5.2. Determination of the empirical transmission rate

The empirical transmission rate *β*_*t*_ for each generation time point by district was used as the dependent variable in a panel regression. From the output of the TSIR model, the empirical transmission rate is calculated as

4.3 $$\beta_{t}=\frac{I_{t+1}N_{t}}{I_{t}^{\alpha }S_{t}},$$

where *I* denotes the number of infected individuals, *S* denotes the number of susceptible individuals, *N* denotes the district population and *α* is a constant that captures heterogeneities in population mixing and the discretization of a continuous time process. *S*, *α* and the reporting rate *ρ*, which allows for a corrected estimate of the true case numbers, are reconstructed from the district-specific output of the TSIR model. The time period separating time points *t* and *t* + 1 is the generation time of dengue, which is taken to be two weeks [2]. For certain time steps, the estimated values of *β*_*t*_ were undefined depending on the corresponding values of infected individuals *I* at the relevant time points *t* and *t* + 1. In a first instance to mitigate this effect, the transmission rate was assigned a value of *β*_*t*_ = 0 when the number of infected individuals at the next time point was zero (*I*_*t*+1_ = 0). Furthermore, following the determination of the empirical transmission rate time series, undefined values of *β*_*t*_ were replaced with either the mean value of surrounding finite values of *β* or simply *β*_*t*−1_, depending on the position of *t* in the panel time series.

When constructing the panel regression model, districts for which the *R*^2^ value associated with the linear regression of the transmission rate time series derived from the TSIR model against the empirical transmission rate time series was *R*^2^ ≤ 0.5 were omitted (three districts). These districts (Kilinochchi, Mannar and Mullaitivu; indicated as the shaded areas in the electronic supplementary material, figure S4) share the common feature of low case numbers, and resulted in empirical transmission rates with high variance and irregular temporal dynamics.

#### 4.5.3. Determination of the lag period for climate variables

Unlike the directly transmitted infection varicella considered in [43], the vector-borne nature of dengue implies that its transmission dynamics depend on prior as opposed to contemporary climatic conditions. In order to estimate the correct lag for the climate variables considered here, for each of the districts retained in the regression model, the empirical transmission rate time series were cross-correlated against each the average temperature and precipitation time series (interpolated to also be structured at time points coinciding with the generation time of dengue) for the pre-epidemic years of 2010–2016. Lag times of up to one year were considered in each cross-correlation, and only such that the climate variables led the empirical transmission rate. Using a peak finding algorithm, the local maxima and minima of the cross correlation function were subsequently located, and statistically significant values retained. Finally, from this subset, the shortest lag time corresponding to a local cross-correlation maximum or minimum was selected as the appropriate lag for that district and climate variable. These lag values are shown for all districts retained in the regression model in the electronic supplementary material, figure S4, for both average temperature (figure S4*a*) and precipitation (figure S4*b*). Note that the same three districts omitted from the panel regression model were also excluded from these calculations as a result of the irregular temporal dynamics of their empirical transmission rate time series.

Once the appropriate district-level lag value was determined for each climate variable, the national lag value was chosen as the most frequently occurring district-level lag. For the subsequent regression model, the climate variable-specific *national* lag value was used for all districts. In practice, for the present-day reanalysis climate data, this lag was implemented by shifting the time series in order to include data from the previous year. For the future climate projections over the two 20 year time periods, the lag was implemented by rotating the annual cycle by the appropriate delay period. In the case of average temperature, a 10 week lag was found. This value is in reasonable agreement with the 10 week lag between temperature and *incidence* assumed in [33], which was selected to account for a six week lag between temperature and oviposition based off findings for *Aedes aegypti* in Ecuador [13] as well as an assumed four week period to account for transmission, disease development, medical-care seeking, and case reporting. In the case of precipitation, a four week lag was found, which again is in reasonable agreement with the lag of three weeks between precipitation and ovitrap data in [13]. Although beyond the scope of the present work, we note that several disease transmission models explicitly include climate-driven vector life cycle dynamics, and for examples of such work the interested reader is referred to [44,45].

#### 4.5.4. Development of the panel regression model

Finally, the regression panel was constructed with log(*β*_*t*_ + 1) as the dependent variable, and the appropriately lagged climate parameters as the dependent variables, yielding the regression model

4.4 $$\begin{array}{l} \log\left(\beta_{t,i}+1\right)=b_{1}f({\text{Tavg}}_{\mathrm{lag},t,i})+b_{2}f({\text{Precip}}_{\mathrm{lag},t,i})+\gamma_{i,m}\\ \;\;\;+\delta_{i,y}+\epsilon_{t,i}, \end{array}$$

where *t* denotes the time and *i* denotes the district. Location-by-month dummies (*γ*_*i*,*m*_) are included to remove location-specific seasonal variation in transmission which may be confounded by other seasonally varying factors. Location-by-year dummies (*δ*_*i*,*y*_) are included to remove location-specific trends in transmission that may be spuriously correlated with climate. Standard errors were clustered at the district level.

In order to determine appropriate functional forms for the climate variables in equation (4.4), non-parametric binned models of the lagged average temperature and precipitation were constructed. The results from these models with 95% confidence intervals for precipitation and average temperature are shown in the electronic supplementary material, figures S5*a* and S5*b*, respectively.

Although precipitation is critical in the mosquito life cycle for the provision of breeding sites as well as the aquatic larval and pupal stages, its quantitative effect on vector viability is less well established. By contrast, numerous studies have experimentally determined the effect of average temperature on various biological traits of mosquitoes and, when appropriate for the disease in question, parasites or viruses [6,33,46–49]. In general, mosquito and virus traits that drive disease transmission exhibit non-monotonic thermal responses, increasing initially as a function of temperature before decreasing, following peak trait performance at a critical temperature value [33].

Motivated by this, we fitted the temperature response to a Brière function:

4.5 $$f_{\mathrm{Brière}}(\text{Tavg})=c\text{Tavg}(\text{Tavg}-T_{\min}){\left(T_{\max}-\text{Tavg}\right)}^{1/2},$$

following [33]. Caveats to this fitted selection are discussed in §2.5. Next, we fitted equation (4.5) to the data in the electronic supplementary material, figure S5b, assuming that above the fitted maximum and minimum temperatures there is no effect of average temperature on log transmission. We impose a value of *T*_max_ = 38.63°C, the average value of the fitted maximum temperature values for all *A. aegpyti* traits reported in the electronic supplementary material, table B of [33], and obtain parameter estimates of *c* = 4.99 × 10^−4^ and *T*_min_ = 25.27°C from this fit. The resulting curve is shown in the electronic supplementary material, figure S5*c* along with the filled circles corresponding to the temperature response points from the binned model.

Finally, we obtain two possible regression models for log-transmission rate on the lagged climate variables of interest by considering either linear responses in both average temperature and precipitation (i.e. *f*(Tavg_lag,*t*,*i*_) = Tavg_lag,*t*,*i*_ and *f*(Precip_lag,*t*,*i*_) = Precip_lag,*t*,*i*_), or a Brière response for average temperature and a linear response for precipitation (i.e. $f({\text{Tavg}}_{\mathrm{lag},t,i})=f_{\mathrm{Brière}}({\text{Tavg}}_{\mathrm{lag}})$ (equation (4.5)) and *f*(Precip_lag,*t*,*i*_) = Precip_lag,*t*,*i*_). The regression tables for both models are shown in the electronic supplementary material, table S1.

Because the predicted future values of average temperature generally lie below the critical thermal response temperature (see the electronic supplementary material, figures S2 and S5*c*), both models yield similar predictions for the effect of future average temperature on log transmission (see the electronic supplementary material, figure S6). The predictions for the district of Nuwara Eliya provide an interesting exception to this pattern, as discussed in §2.5. Ultimately, because of the more realistic prediction of the thermal response of the biological traits provided by the Brière temperature function, the first regression model of a linear response in precipitation and a Brière function for average temperature is used in the analysis of the main text.

### 4.6. Estimation of the fractional increase in susceptible individuals at the onset of serotype invasion for the two-strain susceptible-infected-recovered model

We consider a simplified two-strain system based on that proposed in [23], shown schematically in the electronic supplementary material, figure S1*b*, in order to study the possibility of a population-level susceptibility increase owing to serotype invasion. Let *S* denote the category of individuals susceptible to infection by both strains, *I*_1_ and *I*_2_ denote individuals with primary infections of serotype 1 or 2, respectively, and *R*_1_ and *R*_2_ denote individuals recovered from primary infections with serotypes 1 or 2, respectively. *I*_12_ and *I*_21_ denote individuals previously recovered from infections with serotypes 1 or 2, now experiencing a secondary infection with serotypes 2 or 1, respectively, and *R* denotes the class of individuals recovered from secondary infections. The total number of individuals is *N*. The birth and death rate are denoted by *μ*, and *γ* is the recovery rate from infection. Finally, *ρ*_1_ and *ρ*_2_ denote the importation rate of exogenous infections of serotype 1 and 2, respectively. Additionally, we make a few simplifying assumptions compared to the model described in [23]. First, we assume that the transmission rate *β* is identical for primary and secondary infections, implying that *R*_0_, or the number of secondary infections arising from a single primary infection in an entirely susceptible population, is identical in both cases [50]. This assumption still allows for the possibility of antibody dependent enhancement, or facilitated within-host replication of a heterologous viral strain upon secondary infection as a result of cross-reactive antibodies generated by a previous exposure [51]. Furthermore, the assumption of a uniform *R*_0_ has been made previously in two-strain models of DENV infection [51], and it has been demonstrated that complex transmission dynamics can be achieved without asymmetries in *R*_0_ when effects of temporary cross-immunity between heterologous strains are considered [52]. As an additional modification to the model in [23], we ignore short periods of cross-immunity as we are primarily concerned with the initial conditions at serotype invasion as opposed to the longer-term dynamics.

Prior to the re-emergence of serotype 2, the ordinary differential equations governing this mass action model are

4.6 $$\frac{\text{d}S}{\text{d}t}=-\frac{\beta }{N}SI_{1}+\mu (N-S),$$

4.7 $$\frac{\text{d}I_{1}}{\text{d}t}=\frac{\beta }{N}SI_{1}-(\gamma +\mu )I_{1}$$

4.8 $$\;\mathrm{and}\;\frac{\text{d}R_{1}}{\text{d}t}=\gamma I_{1}-\mu R_{1}.$$

At steady state, these equations yield S* = *N*/*β*(*γ* + *μ*), $I_{1}^{*}=\mu N/(\gamma +\mu )[1-(\gamma +\mu )/\beta ]$, and $R_{1}^{*}=\gamma N/(\gamma +\mu )$ [1−(γ+μ)/β].

At the moment of importation of *ρ*_2_ *N* serotype 2 infections, the governing differential equations become

4.9 $$\frac{\text{d}S}{\text{d}t}=-\frac{\beta }{N}S(I_{1}+\rho_{2}N)+\mu (N-S),$$

4.10 $$\frac{\text{d}I_{1}}{\text{d}t}=\frac{\beta }{N}SI_{1}-(\gamma +\mu )I_{1},$$

4.11 $$\frac{\text{d}I_{2}}{\text{d}t}=\frac{\beta }{N}S\rho_{2}N,$$

4.12 $$\frac{\text{d}R_{1}}{\text{d}t}=\gamma I_{1}-\mu R_{1}-\frac{\beta }{N}R_{1}\rho_{2}N$$

4.13 $$\;\mathrm{and}\;\frac{\text{d}I_{12}}{\text{d}t}=\frac{\beta }{N}R_{1}\rho_{2}N.$$

Consequently, gathering terms from equations (4.9)–(4.13) associated with the generation of newly infected individuals, we see that the total rate of generation of new infections at this time point is given by

4.14 $$\frac{\beta }{N}[SI_{1}+(S+R_{1})\rho_{2}N],$$

which can be re-expressed as the product of the population-normalized transmission rate and the total number of susceptible and infected (*I*_1_ + *ρ*_2_*N*) individuals at the previous time step. Doing so we obtain

4.15 $$\frac{\beta (I_{1}+\rho_{2}N)}{N}\left[S+R_{1}\left(1-\frac{1}{1+\frac{\rho_{2}N}{I_{1}}}\right)\right].$$

Assuming the number of imported cases is small relative to the number of autochthonous infections (i.e. *ρ*_2_*N* ≪ *I*_1_), the expression in equation (4.15) simplifies to

4.16 $$\frac{\beta (I_{1}+\rho_{2}N)}{N}\left[S+\frac{R_{1}\rho_{2}N}{I_{1}}\right].$$

Recognizing that at this time point $R_{1}=R_{1}^{*}$, $I_{1}=I_{1}^{*}$ and *S* = S* from the single strain equilibrium result, the number of susceptible individuals at the time of importation of serotype 2 infections can be expressed as

4.17 $$S^{\prime }\approx S+\frac{R_{1}\rho_{2}N}{I_{1}}\approx S^{*}\left[1+\frac{\rho_{2}\gamma \beta }{\mu (\gamma +\mu )}\right].$$

## Data accessibility

All data used in this paper are publicly available as described in §§4.1–4.4.

## Code availability

All numerical code used for simulations and analysis can be found at https://github.com/cewagner/Dengue-Sri-Lanka.
